# An Australian translational Study to evaluate the prognostic role of inflammatory markers in patients with metastatic ColorEctal caNcer Treated with bevacizumab (Avastin™) [ASCENT]

**DOI:** 10.1186/1471-2407-13-120

**Published:** 2013-03-15

**Authors:** Stephen Clarke, Matt Burge, Cassandra Cordwell, Peter Gibbs, William Reece, Niall Tebbutt

**Affiliations:** 1 Royal North Shore Hospital, St Leonards, NSW 2065, Australia; 2 Royal Brisbane and Women Hospital, Butterfield, QLD, Australia; 3 Roche Products, Pty. Limited (Australia), Dee Why, NSW, Australia; 4 Royal Melbourne Hospital, Parkville, VIC, Australia; 5 Covance Pty Ltd, Sydney, NSW, Australia; 6 Austin Health, Heidelberg, VIC, Australia

**Keywords:** Colorectal cancer, Bevacizumab, Treatment beyond progression, Biomarkers, Inflammation

## Abstract

**Background:**

The use of bevacizumab in combination with fluoropyrimidine-containing chemotherapy is a well-established first-line and second-line treatment for patients with metastatic colorectal cancer (mCRC). However, there remains a need for reproducible, validated, inexpensive and accessible prognostic markers to aid treatment selection. The optimal treatment duration and the role of bevacizumab in certain patient subgroups, considered at particular risk of bevacizumab-mediated toxicity, also require further investigation. The aim of the ASCENT study [an Australian translational Study to evaluate the prognostic role of inflammatory markers in patients with metastatic ColorEctal caNcer Treated with bevacizumab (Avastin™)] is to evaluate the relationship between the host inflammatory response as measured by neutrophil/lymphocyte ratio (NLR) and treatment outcomes in patients with previously untreated mCRC receiving bevacizumab-based first- and second-line treatment.

**Methods/design:**

This open-label, prospective, single arm, phase IV, Australian multi-centre study evaluates the relationship between the host inflammatory response as measured by NLR and treatment outcomes in patients with previously untreated mCRC receiving bevacizumab-based first- and second-line treatment. 150 patients will be recruited from 16 centres around Australia. Patients will receive trial treatments in two phases: *Phase A*: XELOX or mFOLFOX6 plus bevacizumab administered from study start until first disease progression; and *Phase B*: FOLFIRI plus bevacizumab administered from first disease progression until second disease progression. The primary analysis will test the association between NLR and progression free survival using a proportional Hazards Model. Secondary analyses will investigate whether the relationship can be improved upon with other prognostic biomarkers, and further characterise the safety of bevacizumab following treatment initiation, and when continued after progression in combination with standard chemotherapy regimens (presented through summary statistics and Kaplan Meier curves).

**Discussion:**

Quantifying the relationship between NLR and PFS will inform decision making on the extent to which this simple metric may be applied clinically.

**Trial registration:**

ClinicalTrials.gov: NCT01588990

## Background

Colorectal cancer (CRC) is the second most commonly diagnosed cancer in Australia, with 14300 new cases and 4047 deaths (2,191 males; 1,856 females) [1] recorded in 2007. It is projected that 19000 new cases of CRC will be diagnosed, in Australia, in 2020 [2]. Approximately 25% of patients present with metastatic CRC (mCRC) at initial diagnosis and another 25% will develop subsequent metastases [3]. Treatment outcomes have improved significantly in the last decade as a result of the introduction of new systemic treatments and the expanded use of hepatic metastatectomy; with median survivals now well in excess of two years [4].

For the majority of patients diagnosed with mCRC palliative chemotherapy is the most appropriate treatment option in order to achieve the goals of prolonging survival and improving quality of life (QoL). The backbone of first- and second-line palliative chemotherapy for mCRC consists of a fluoropyrimidine (infusional 5-FU or oral capecitabine) based therapy in various combinations and schedules. Combination chemotherapy with fluoropyrimidine/oxaliplatin (FOLFOX or XELOX) or 5-FU/LV/irinotecan (FOLFIRI) provides higher response rates, longer progression-free survival (PFS) and better overall survival (OS) than a fluoropyrimidine alone. Both FOLFOX/XELOX and FOLFIRI have similar efficacy regardless of the sequence used but have different toxicity profiles [5]. Favorable survival has been shown to correlate with the percentage of patients receiving all active chemotherapeutic agents, emphasizing the importance of exposure to all active drugs during treatment [6].

The use of bevacizumab in combination with fluoropyrimidine-containing chemotherapy is a well-established first-line and second-line treatment for patients with mCRC [7-13]. Despite this, a number of data gaps remain to be addressed, notably, the need for reproducible, validated, inexpensive and easy to administer prognostic biomarkers to aid in treatment selection. The optimal treatment duration and the role of bevacizumab in certain patient subgroups, specifically those considered at particular risk of bevacizumab-mediated toxicity, also require further investigation.

An increasing proportion of patients with mCRC at first presentation are treated with systemic chemobiologic therapy without pre-emptive resection of the primary tumour. Limited data currently exist to guide treatment decisions in this setting and uncertainty exists around the risk/benefit of bevacizumab-based treatment in patients with a primary *in situ* tumour [14-16]. Although the recent NSABP C-10 trial contributed important data regarding bevacizumab use in the setting of an asymptomatic colonic primary tumour [14], similar studies have not yet been undertaken in patients with a minimally symptomatic primary colon cancer or those with an *in situ* primary rectal cancer. It is therefore necessary to further study the safety and effectiveness of bevacizumab in the setting of an *in situ* primary rectal lesion.

A wealth of preclinical models support the notion that vascular endothelial growth factor (VEGF) is continually expressed throughout the lifecycle of the tumour and that sensitivity to anti-VEGF therapy remains even after disease progression [17]. The continuation of bevacizumab after disease progression on bevacizumab-based first-line treatment (bevacizumab beyond progression or BBP) is common practice in countries such as the United States [18]. Multivariate analyses from two large observational cohort studies (BRiTE and ARIES registries) [19,20] suggest that BBP is an independent predictor of prolonged survival in mCRC. Although the use of BBP has been addressed in a recently published, randomized phase III trial (ML18147) [21], this study was not open in Australia and did not collect data on QoL.

Biomarkers play an increasingly important role in both cancer research and clinical practice. They can be used to assess prognosis and to predict how individual patients will respond to specific treatments [22,23]. Despite concerted international research efforts, there has not yet been a validated and easy to administer biomarker to predict treatment outcomes for patients treated with bevacizumab. A broad range of blood- and tumour tissue-based markers have been explored during the development phase of bevacizumab (preclinical > 10,000; clinical > 100) with most of the existing data focused on VEGF pathway markers, including tumour VEGF expression [24], or oncogene mutations such as K-Ras [25,26]. Relatively little attention has been paid to the role of biomarkers associated with the tumour microenvironment and host factors such as the inflammatory response. Both the tumour microenvironment and the inflammatory response are considered key aspects of cancer biology and tumourigenesis [27] and are important regulators of angiogenesis. Infiltration of small tumours by inflammatory cells that produce proangiogenic ligands makes a contribution to the angiogenic switch that drives tumour growth. Tumour development and progression induced by an inflammatory response is thought to be mediated by pro-inflammatory cytokines stimulating pathways especially those mediated by the nuclear factor kappa-light-chain-enhancer of activated B cells (NF-κB) and the Signal transducer and activator of transcription 3 (STAT3) [28]. Given the established link between systemic inflammation and tumour angiogenesis the potentially valuable role of inflammatory markers as predictive or prognostic tools in the setting of bevacizumab is of interest. The use of blood-based inflammatory markers such as neutrophil/lymphocyte ratio (NLR) as prognostic/predictive biomarkers in patients receiving bevacizumab-based chemotherapy has not yet been evaluated.

Elevated NLR (> 5) has been shown to be predictive of diminished survival in patients with liver-only colorectal metastases receiving neo-adjuvant chemotherapy prior to hepatic metastectomy [29]. Patients in whom NLR normalised after chemotherapy had significantly improved 1-, 3- and 5-year survival which was similar to patients with NLR ≤ 5 at baseline [29]. More recently, a study by Chua *et al.*[30] demonstrated an elevated NLR, pre-treatment, in patients with unresectable mCRC, in approximately 30% of patients. In this patient cohort, who underwent first-line combination chemotherapy, NLR was found to be an independent predictor of clinical benefit, progression and survival. The NLR was statistically significantly associated with overall survival (P < 0.0001). Patients with NLR ≤ 5 had median overall survival of 19.1 months (95% CI 15.3–22.8) compared with patients with NLR > 5 (11.3 months; 95% CI 8.3–14.3). In addition, normalization of the NLR after one cycle of chemotherapy was associated with improved progression free survival.

The primary objective of this study (NCT01588990; ML25753) is to validate and quantify the prognostic value of the host inflammatory response as assessed by the NLR on Progression Free Survival. Secondary objectives include firstly, investigating the relationship further in light of other clinical and biological markers; secondly, providing clinically relevant information regarding the safety, effectiveness and QoL outcomes prior to, and after, progression. Patients will be treated with bevacizumab in combination with standard chemotherapy regimens in a generalized, community-based population of mCRC patients. The study has started recruitment in 16 centres around Australia.

## Methods/design

### Ethics approval

Participating patients will provide written informed consent. The study will be conducted in accordance with local guidelines and in line with the principles of the Declaration of Helsinki and Good Clinical Practice Guidelines. Ethics approval has been obtained from all participating institutions.

### Study objectives

The primary objective of the study is to assess the prognostic value of the host inflammatory response as assessed by the NLR (≤ 5 versus > 5) on Progression Free Survival. The secondary objectives are to further characterise the safety profile of study treatment and evaluate its efficacy following treatment initiation, initial response and when continued after progression; to explore the role of NLR as a predictor of OS in patients treated with bevacizumab; to assess the association between post-baseline changes in NLR and PFS and OS and NRL; to assess patient reported QoL; and to assess the incidence of serious adverse events related to the primary tumour in the primary *in situ* patient cohort.

Exploratory objectives include further characterization of the relationship between blood-based markers of systemic inflammation [including liver-derived acute phase proteins, NLR and platelet/lymphocyte (PLR) ratios and the modified Glasgow Prognostic Score (mGPS)] and standard biochemical parameters (including adjusted calcium, bilirubin, alkaline phosphatase, aspartate transaminase, alanine transaminase and γ-glutamyl transferase) and therapeutic outcomes.

### Study design

**Figure 1:**
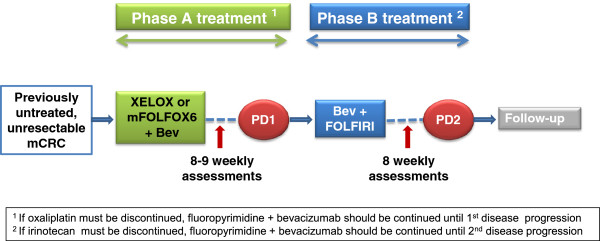
**Study design.** Bev = bevacizumab. PD = progressive disease.

This is an open-label, prospective, single arm, phase IV, Australian multi-centre study evaluating the relationship between the host inflammatory response as measured by NLR and treatment outcomes in patients with previously untreated mCRC who will receive bevacizumab-based first- and second-line treatment (trial design is illustrated in Figure 1). The trial consists of two phases of treatment:

*Phase A treatment*: XELOX or mFOLFOX6 plus bevacizumab administered from study start until first disease progression;

*Phase B treatment*: FOLFIRI plus bevacizumab administered from first disease progression until second disease progression.

Bevacizumab infusions will be administered on a three-weekly basis in combination with XELOX or on a two-weekly basis in combination with mFOLFOX6 throughout Phase A treatment until first disease progression or occurrence of unmanageable toxicity. Upon documented disease progression, Phase A treatment will be discontinued and bevacizumab will be continued on a two-weekly basis in combination with FOLFIRI (Phase B treatment) until second disease progression or unmanageable toxicity. Upon second disease progression, all study treatment will be discontinued and patients will enter follow-up for survival status and subsequent treatment for their mCRC. Phase B treatment (bevacizumab plus FOLFIRI) will commence within 4 weeks of the date of documented first disease progression.

### Investigational product

Bevacizumab administered beyond first disease progression (Phase B) is considered to be the “investigational study drug”. Bevacizumab administered as Phase A treatment is considered to be standard-of-care “non-investigational drug”. XELOX, mFOLFOX6 and FOLFIRI are considered standard of care “non-investigational combination drug”. Collectively, they will be known as the ‘study treatment’.

The doses and regimens of XELOX and mFOLFOX6 and FOLFIRI, administered throughout study Phase A and Phase B, are according to the local Australian treatment recommendations and requirements. In Phase A treatment, bevacizumab will be administered at a dose of either 7.5 mg/kg iv to coincide with XELOX (where bevacizumab will be administered every 3 weeks) or 5.0 mg/kg with mFOLFOX6 (where bevacizumab will be administered every 2 weeks). The dose and regimens of bevacizumab administered throughout Phase A are per local recommendations and requirements. In Phase B treatment, bevacizumab will be administered at a dose of 5.0 mg/kg iv on day 1 every 2 weeks in combination with FOLFIRI. The doses of bevacizumab in Phase B are in line with the dose used in the Phase III study (ML18147) and the observational studies, BRiTE and ARIES [19-21]. The drug doses for each treatment phase are summarized in Table 1.

**Table 1:** Study treatment doses

	**Phase A**	**Phase B**
**XELOX**	Oxaliplatin: 130 mg/m^2^ iv day 1	
**Every 3 weeks**	Capecitabine: 1000 mg/m^2^ po twice daily days 1 to 14	
	Bevacizumab: 7.5 mg/kg iv day 1	
**mFOLFOX6**	Oxaliplatin: 85 mg/m^2^ iv day 1	
**Every 2 weeks**	Leucovorin*: 400 mg/m^2^ iv day 1	
	Fluorouracil: 400 mg/m^2^ iv day 1	
	Fluorouracil: 2400 mg/m^2^ continuous iv infusion over 46 hours day 1	
	Bevacizumab: 5.0 mg/kg iv day 1	
**FOLFIRI**		Irinotecan**:** 180 mg/m^2^ iv day 1
**Every 2 weeks**		Leucovorin*: 400 mg/m^2^ iv day 1
		Fluorouracil: 400 mg/m^2^ iv day 1
		Fluorouracil: 2400 mg/m^2^ continuous iv infusion over 46 hours day 1
		Bevacizumab: 5.0 mg/kg iv day 1

* Investigators may elect to use low dose leucovorin i.e. either 20 mg/m^2^ or 50 mg total dose.

### Patient population and eligibility criteria

The target population for this study includes male and female adult patients with histologically confirmed mCRC eligible to commence first-line treatment with bevacizumab in combination with XELOX or mFOLFOX6. Inclusion and exclusion criteria are presented in Table 2.

**Table 2:** Eligibility criteria


**Inclusion criteria**	
**Resected primary tumour population**	
1.	Signed informed consent obtained prior to any study specific procedures and willingness to comply with study requirements (including biomarker sampling and tumour sampling for biomarkers).
2.	Patients must be ≥ 18 years old.
3.	Histologically confirmed, previously untreated mCRC and not a candidate for curative resection.
4.	WHO performance status of 0–1.
5.	Life expectancy of ≥ 3 months.
6.	Eligible for XELOX, mFOLFOX6, FOLFIRI and bevacizumab treatment in accordance with local standards of care and guidelines.
**Patients with primary tumour *in situ***	
*Resected primary tumour population inclusion criteria apply to the following criteria:*	
1.	Intact primary tumour of the colon or rectum not requiring surgical intervention prior to commencing chemotherapy.
2.	Minimally or asymptomatic primary tumour (without obstruction, perforation or active bleeding requiring transfusion).
**Exclusion criteria**	
**Resected primary tumour population**	
1.	Previous chemotherapy for mCRC.
2.	Previous neoadjuvant or adjuvant chemotherapy completed within 6 months prior to commencement of study treatment.
3.	Radiotherapy within 28 days prior to enrolment or from which patients have not yet recovered.
4.	History of non-colorectal cancer (patients are eligible if they have been disease-free for ≥ 5 years and the risk for recurrence is deemed low).
5.	Presence of active inflammatory bowel disease.
6.	History of gastrointestinal perforation.
7.	Symptomatic or bulky peritoneal disease.
8.	History of significant bleeding event(s).
9.	Significant vascular disease.
10.	Peripheral arterial thrombosis or other thrombotic event within 6 months prior to commencement of study treatment.
**Patients with primary tumour *in situ***	
*Resected primary tumour population exclusion criteria apply in addition to the following criteria:*	
1.	Prior endoscopic management of the current malignancy.
2.	Acute diverticulitis.
3.	Presence of intra-abdominal abscess.
4.	Active gastroduodenal ulcer(s).

### Assessments and procedures

Patients who provide informed consent will be screened 7–14 days before the baseline visit. Patients who fulfill all of the inclusion and none of the exclusion criteria will be accepted into the study. Treatment will be commenced within 7 days of the baseline visit. Patients will attend study specific visits every 8 or 9 weeks (to coincide with chemotherapy regimen) throughout Phases A and B. All patients will undergo a safety assessment no later than 30 days after the last dose of study treatment in Phase A, and an end of treatment (EoT) safety assessment no later than 30 days after the last dose of study treatment in Phase B. Patients will have subsequent follow-up visits every 12 weeks until study end. At the study end, all patients will have an end of study follow-up visit to evaluate progression, survival status and safety. And patients, who have discontinued study treatment for reasons other than progressive disease whilst in either Phase A or Phase B, will enter follow-up.

All data for secondary outcomes will be collected on a case record form (CRF) by the treating physician. Data regarding QoL (EroQol-5-D, AQol-8D and FACT-C) will be captured using self-reported questionnaires, at baseline, during treatment period, and safety and survival follow-up visits.

### Statistical considerations and analytical plan

#### Sample size

Approximately 150 patients will be enrolled into the study (approximately 105 resected primary tumour population patients and approximately 45 primary *in situ* tumour patients) or recruitment will cease after 24 months, whichever occurs first. The sample size was determined based on the assumptions that the true incidence of NLR > 5 is 30%, the median PFS is 10.5 months in patients with NLR ≤ 5, and all patients are followed for 24 months. The incidence of NLR > 5 of 30% is based on the values reported by Chua *et al*[30].

This provides approximately 80% power to detect a hazard ratio of 1.7 for the effect of NLR on PFS in the primary endpoint. It is anticipated that the hazard ratio for NLR in the primary model will be larger than the hazard ratio (1.6) observed in the multivariate analysis reported by Chua *et al.*[30]. This is because the multivariate analysis adjusted for the presence of hypoalbuminemia, which is likely to be correlated with NLR, and would have reduced the apparent association.

#### Analysis populations

The Full Analysis Set will include all patients who receive at least one dose of bevacizumab. The “Primary *In Situ* population” will include all patients in the Full Analysis Set with a primary *in situ* tumour. The “Resected Primary Tumour population” will include all patients in the Full Analysis Set without a primary *in situ* tumour.

#### Statistical analysis

The primary analysis will be a Cox Proportional Hazards model of baseline NLR (< 5 vs. ≥5) on Progression Free Survival, adjusted for WHO performance status, presence of metastatic disease in the liver, number of different sites of metastatic disease, and presence of metastatic disease in the liver with no other sites of involvement. Secondary relationship analyses will build on this primary model by adding or removing predictors. Safety analyses will be descriptive with no pre-defined hypotheses.

### Recruitment and participating sites

Sixteen centres across Australia will participate in the study.

### Time-line for the study

The study has started recruitment in June 2012 and will formally end 24 months after the date of the commencement of treatment for the last patient enrolled or once all patients have died or have withdrawn from the study, whichever occurs first, but may be prematurely terminated by the sponsor.

## Discussion

To date there is no validated or reproducible prognostic biomarker to assist clinicians with determining the most likely treatment outcomes for patients with mCRC treated with bevacizumab-containing regimens. Relatively little attention has been paid to the role of host/tumour microenvironment factors such as the inflammatory response and whether anti VEGF therapy might be able to abrogate an inflammatory microenvironment that is favourable to tumour growth/metastasis. The influence of the host inflammatory response, as measured by NLR, has not yet been studied in the setting of bevacizumab and may represent a clinically useful prognostic marker. This study will provide important data to clarify the role of NLR as a prognostic factor in the setting of standard first-and second-line therapy for mCRC. Due to the single arm design of the study, NLR as a predictor of response to bevacizumab cannot be established.

Secondary objectives of this study will further characterise the safety and efficacy of bevacizumab beyond progression in combination with standard chemotherapy regimens in a more generalised, community-based population of mCRC patients in Australia and will evaluate treatment outcomes of approximately 45 patients presenting with mCRC with a minimally symptomatic or asymptomatic primary colon or rectal tumour.

## Abbreviations

BBP: Bevacizumab beyond progression; CRC: Colorectal cancer; CRF: Case report form; EGFR: Epidermal growth factor receptor; EoT: End of treatment; FOLFIRI: Infusional 5-fluoarouracil, leucovorin and irinotecan; mCRC: Metastatic colorectal cancer; mFOLFOX6: Modified infusional 5-fluorouracil, leucovorin and oxaliplatin (2 weekly schedule); NLR: Neutrophil/Lymphocyte ratio; NYHA: New York Heart Association; OS: Overall survival; QoL: Quality of life; VEGF: Vascular endothelial growth factor; WHO: World health organization; XELOX: Oral capecitabine plus infusional oxaliplatin.

## Competing interests

SC, MB, and NT did not declare any competing interest. CC is an employee of Roche Products Pty Limited. PG has participated in Roche advisory boards. WR is an employee of Covance Pty Ltd who is contracted to Roche Products Pty Limited to support this research.

## Authors’ contributions

SC is the principal investigator on the study and was involved in study design, protocol development and preparation of the manuscript. MB, CC, PG and NT are members of the study steering committee and were involved in the study design, protocol development and the review of the manuscript. WR is the study statistician and has been involved in study design, the development of the analysis plans and the development and review of the manuscript. All authors read and approved the final manuscript.

## Pre-publication history

The pre-publication history for this paper can be accessed here:

http://www.biomedcentral.com/1471-2407/13/120/prepub
